# Distribution of ABO and Rh blood groups among pregnant women attending the obstetrics and gynecology clinic at the Jordan University Hospital

**DOI:** 10.1038/s41598-023-40085-w

**Published:** 2023-08-14

**Authors:** Oqba Al-Kuran, Lama AL-Mehaisen, Rawan Qasem, Saja Alhajji, Nour Al-Abdulrahman, Shaikha Alfuzai, Sara- Alshaheen, Lena Al-Kuran

**Affiliations:** 1 Department of Obstetrics and Gynecology, Faculty of Medicine, Jordan University, Amman, 11972 Jordan; 2 Obstetrics and Gynecology, FRCOG, AL-Balqa Applied University, As-Salt, 19117 Jordan; 3 Faculty of Medicine, Jordan University, Amman, 11972 Jordan

**Keywords:** Health care, Medical research

## Abstract

The ABO and D antigen status of red blood cells (Rh blood grouping systems) are important hematological classification systems that categorize blood groups according to the presence or absence of certain erythrocytic antigens. These antigens affect the outcomes of blood transfusions as well as various hematological and immunological diseases. We aimed to study ABO and Rh blood group distribution among pregnant women visiting the antenatal care clinic at Jordan University Hospital (JUH) in Amman, Jordan. A retrospective analysis of all pregnant women delivering at the Jordan University Hospital (JUH) between October 1, 2016, and September 31, 2021. ABO and D antigen status of red blood cells (Rh blood groups) were summarized and documented. 20,136 pregnant women data were analyzed, the O blood group was the most prevalent (n = 7840, 38.9%), followed by A (n = 7506, 37.3%). For the D antigen status, the Rh-positive (Rh+) category was the most common (n = 18,159, 90.2%). For the (O) blood group; O-Rh+ type was the most prevalent (90.1%). Determining the blood group type accurately helps eliminate the critical consequences of both ABO and Rh incompatibility and offers clinicians an opportunity to take timely prophylactic measures. In our analyses O and Rh+ blood groups were the most prevalent.

## Introduction

The ABO and Rh blood group systems, The ABO blood group system was discovered in 1900 by Karl Landsteiner but the RH system was discovered in 1940 by Landsteiner and Alexander Wiener^1^, form a crucial part of the hematological system in the human body with a significant role in blood transfusion and hematological and immunological diseases. Each person has a different blood type which is determined by the inherited genetic composition from their parents. The major blood groups are categorized into four basic categories: A, B, AB, and O. Each of these blood groups produces group-specific antibodies in the plasma; for instance, people with the A blood group have type-A antigens on their erythrocytes and antibodies against B antigens, which contributes to a process called agglutination. The agglutination indicates that the red blood cells have reacted with a certain antibody but if the blood does not agglutinate, it indicates that the blood does not have the antigens binding the antibody in the reagent.

In summary, blood group A—has A antigens on the red blood cells with anti-B antibodies in the plasma while blood group B—has B antigens with anti-A antibodies in the plasma. Blood group O—has no A or B antigens, but both anti-A and anti-B antibodies in the plasma, and blood group AB—has both A and B antigens, but no antibodies.

A second additional system of classifying blood groups is the Rh system which determines the presence (Rh+) or absence (Rh−) of a protein in an individual’s erythrocytes i.e., Rh+ refers to RhD antigen expression while Rh− refers to a lack of RhD antigen expression. Each of the ABO blood groups may be Rh+ or Rh− based on the presence of Rh antigen on its surface^2^.

A Jordanian retrospective study reported that O and A were the most prevalent blood groups (37.44% and 36.82%, respectively), followed by B (18.62%) and AB (7.12%). The Rh+ ve was more prevalent (88.73%) compared with Rh−ve (11.27%)^3^_._

Another Nigerian retrospective study reported that type O was the most frequent (47.7%), followed by type A (26.6%), type B (22.6%), and AB (3.5%), and 97.1% of the study sample was (Rh+)^4^. Furthermore, the Libyan study reported that the negativity rate for Rh antigen was higher in O and A blood groups (5.83%), followed by the B (3.3%) and AB (0.8%) blood groups^2^. These differences are chiefly related to an individual’s genetic and ethnic factors. Both blood-grouping systems are of great value in transfusion safety, genetic research, inheritance pattern, paternity testing, and susceptibility to certain hereditary diseases, such as duodenal ulcer, diabetes mellitus, urinary tract infections, gastric cancer, and predisposition to lower levels of von Willebrand factor^5^.

Hemolytic disease of the fetus and newborn (HDFN), one of the foremost causes of perinatal morbidity and mortality, is an emergency that occurs most likely during the second pregnancy due to Rh status incompatibility and to a lesser degree due to ABO incompatibility.

When an Rh-negative mother is sensitized to the Rh antigen (from the Rh-positive blood groups) either during previous pregnancies from fetal Rh+ erythrocytes or subsequent incompatible transfusions, she develops Rh antibodies, eventually leading to hemolysis of fetal erythrocytes^4^.

Hemolytic disease of the fetus and newborn (HDFN) affects 3/100 000 to 80/100 000 patients per year^6^.

Maternal antibodies destroy fetal red cells and in some cases lead to bone marrow suppression. This process leads to fetal anemia, and in severe cases can progress to edema, ascites, heart failure, and death. Fetuses with (HDFN), are followed up at the fetal medicine unit at the University of Jordan, and multiple interventions are done depending on the stage of (HDFN), starting with fetal blood sampling and ending with multiple fetal blood transfusions in some cases. HDFN due to RhD can be prevented by Rh immunoglobulin administration.

HDFN can rarely be caused by ABO incompatibility, often less severe than Rh incompatibility^7^, when the mother and fetus have different ABO blood types resulting in maternal antibodies against the fetus’s erythrocytes. This type of incompatibility is most common in mothers having type-O blood group with a fetus that has A, B, or AB blood group; the IgG antibody is responsible for this condition. ABO incompatibility is one of the serious causes of hyperbilirubinemia in the fetus leading to jaundice and anemia in newborns and requiring extensive treatment.

Therefore, comprehensive knowledge of the distribution pattern of different blood types is imperative for the effective prevention and management of the fatal erythroblastosis fetalis by maintaining blood bank reserves and reducing morbidity and mortality due to blood transfusions. Since the data regarding the prevalence of different blood groups in Jordan is limited, we conducted this experiment to evaluate the distribution of ABO and Rh blood groups among pregnant women at the Gynecology and Obstetrics department at the Jordan University Hospital, Jordan. We believe this data will aid health workers in the effective management and prevention of blood group incompatibility-related complications.

Knowing our nation’s blood group distribution will aid in allocating our resources, and assess blood bank protocols.

It would be interesting to run another study a few years from now and compare our results as we are expecting changes due to the immigration waves Jordan has witnessed the last few years.

## Methods

We used a retrospective study design to include a cohort of all pregnant women aged > 18 years who gave birth at the Jordan University Hospital at more than 24 weeks gestation between October 1, 2016, and September 31, 2021. The medical records of the patients were extracted from the hospital’s electronic records and laboratory information system. Results of only the blood group test of the patient were included for analysis.

We obtained ethical approval from the Faculty Scientific Ethical Committee, at Jordan University Hospital. We maintained strict confidentiality throughout the study.

Blood samples were collected from all patients by antecubital vein puncture and were kept in an Ethylene Diamine Tetra Acetic acid (EDTA) anticoagulant bottle and centrifuged at 4000 rpm for 10 min at room temperature. ABO and Rh blood group tests were executed using standard gel centrifugation. The cards used in gel centrifugation have microtubes on each card containing buffered gel solution with specific antibodies, including anti-A, anti-B, anti-AB, anti-D, and anti-C/D/E. Agglutination occurs when the erythrocyte antigen reacts with the corresponding antibodies present in the gel solution or the serum or plasma sample. By using a gel column, agglutinated erythrocytes are captured based on their size. When agglutinated erythrocytes are captured at the top of the gel column, the test is positive. When non-agglutinated erythrocytes reach the bottom of the microtubes, they form a pellet and yield negative results. The data so obtained from the medical records were processed using SPSS (IBM Inc., Armonk, NY, USA) for appropriate statistical analysis. The prevalence of different blood group types is expressed as frequency and percentages.

In our university labs, direct agglutination molecular tests are used for the determination of weak D types. Weak and partial RhD were considered as Rh positive due to our major concern in Rh D-negative pregnant females as it can produce alloimmunization if accidentally given weak/partial RhD antigen-positive blood, knowing that weak D antigen ranges from 0.2 to 1%.^8^

The study was approved by IRB Committee of the faculty of Medicine at the University of Jordan. The need for informed consent was waived by the IRB of the faculty medicine at the University of Jordan. All procedures were performed in accordance with relevant guidelines at the University of Jordan.

### Ethics approval

This study has been approved by the Ethical committee of the University of Jordan. All procedures were performed in accordance with relevant guidelines at the University of Jordan.

### Patient consent

The need for informed consent was waived by the IRB of the faculty medicine at the University of Jordan.

## Results

**Figure 1:**
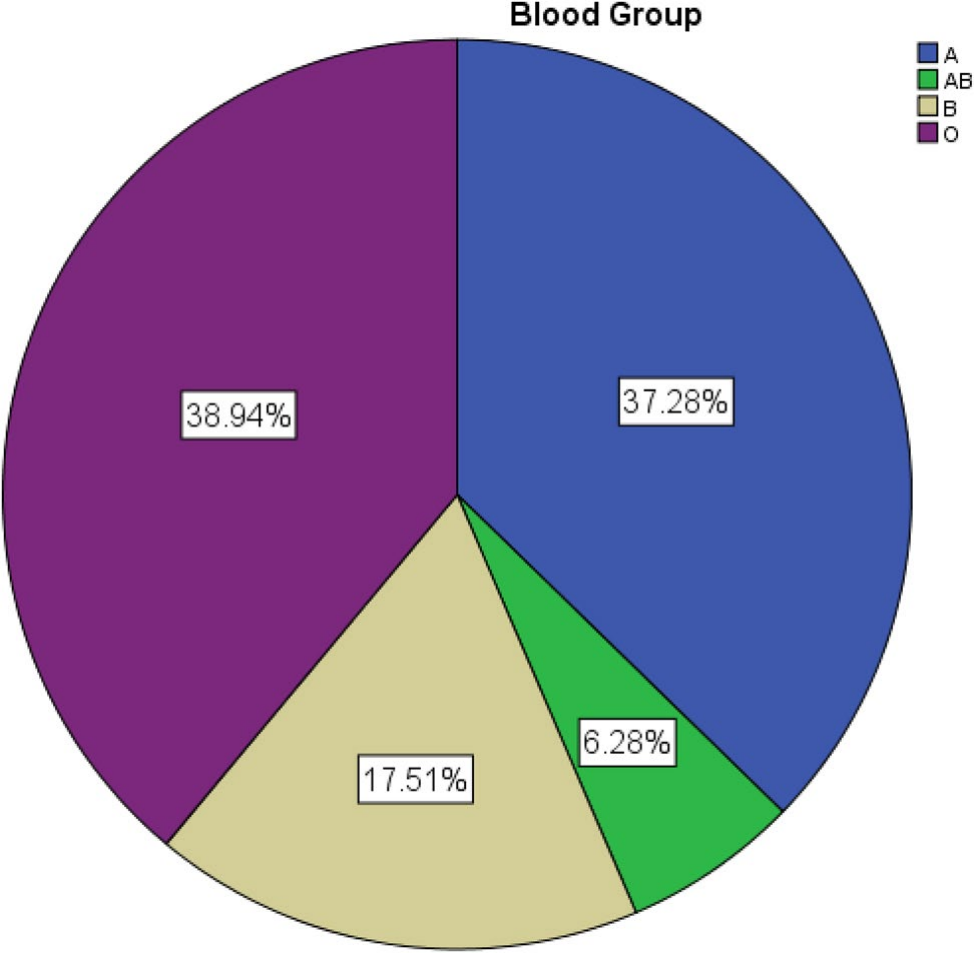
Pie chart for ABO blood groups.

**Figure 2:**
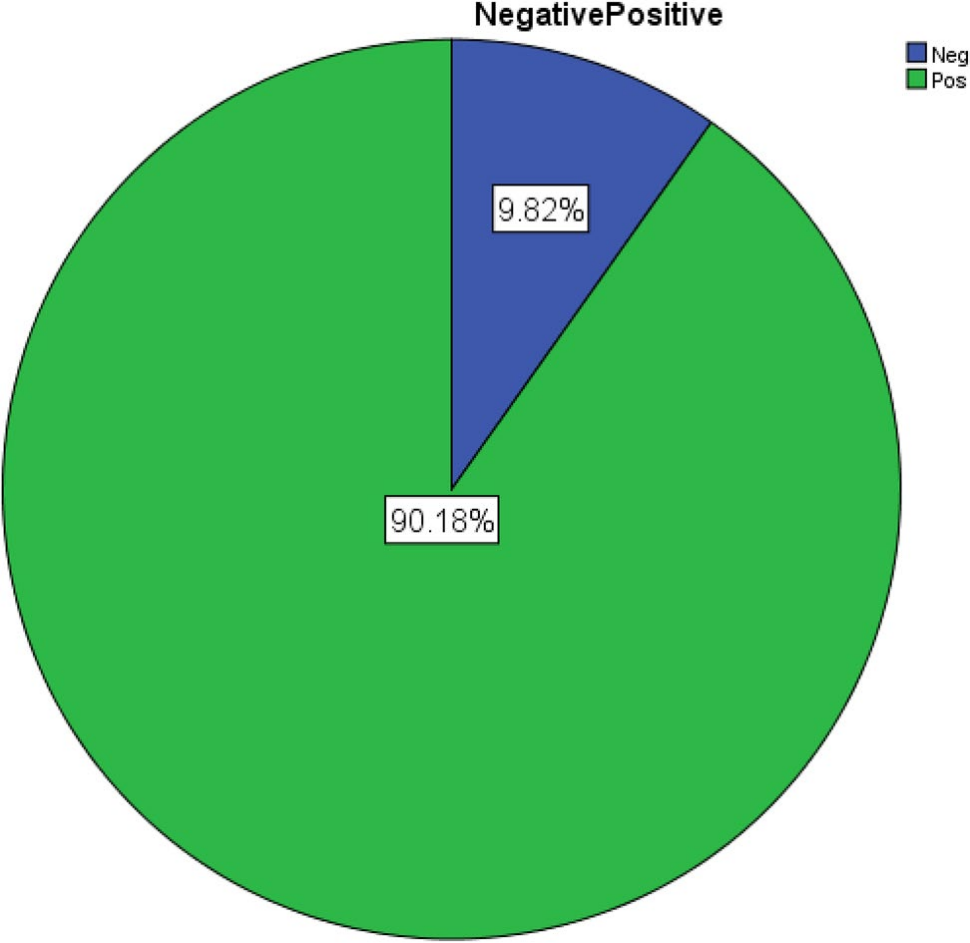
Pie chart for RH groups.

We included data for 20,136 pregnant women in this study. The majority of participants had an O blood group (n = 7840, 38.9%), (Table 1). Regarding the Rh system, Figs. 1 and 2 illustrate the distribution pattern of different blood groups using pie charts.

**Table 1:** Incidence for ABO and Rh blood groups.

Blood group	Categories	Overall n%
ABO type	A	7506 (37.3%)
	AB	1265 (6.3%)
	B	3525 (17.5%)
	O	7840 (38.9%)
Rh	Negative	1977 (9.8%)
	Positive	18,159 (90.2%)

Table 2 presents the association results between the ABO and Rh blood group types.

**Table 2:** Association between ABO and Rh blood groups.

		RH		
		Negative	Positive	Total
ABO Blood Group	A	729 (9.7%)	6777 (90.3%)	7506 (100.0%)
	AB	130 (10.3%)	1135 (89.7%)	1265 (100.0%)
	B	343 (9.7%)	3182 (90.3%)	3525 (100.0%)
	O	775 (9.9%)	7065 (90.1%)	7840 (100.0%)
	Total	1977 (9.8%)	18,159 (90.2%)	20,136 (100.0%)

There was no association between the ABO and Rh blood groups.

**Figure 3:**
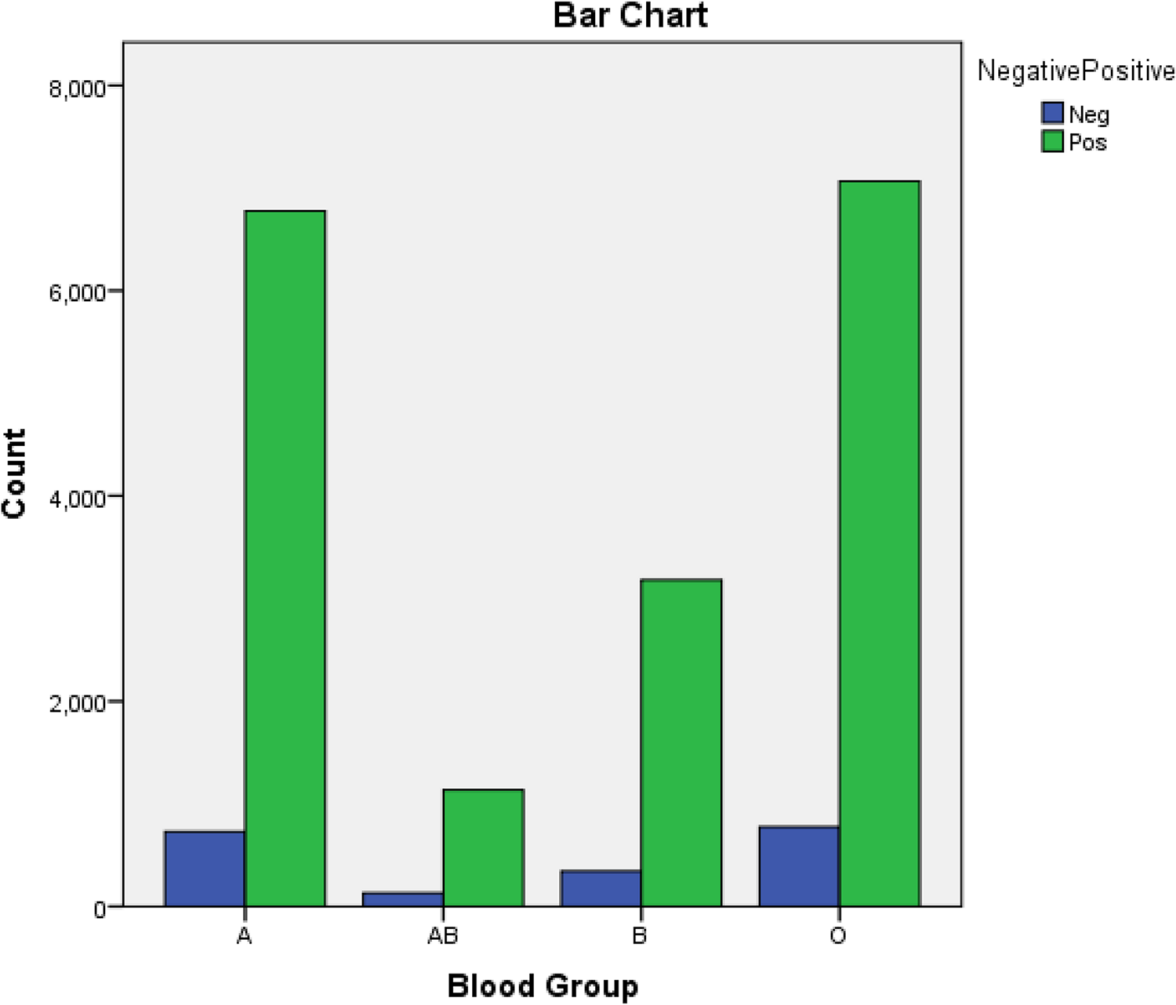
Bar chart for the crosstabulation between ABO blood groups and RH.

The prevalence of A blood group with Rh− type was only 9.7% compared to 90.3% incidence of type A with Rh+. Figure 3 illustrates the association between different ABO and Rh blood groups.

## Discussion

We found that the O type was the most common blood group (38.9%) in Jordanian pregnant women, followed by the A, B, and AB types. Likewise, the Rh+ (D) was the most prevalent blood group (90.2%) compared to only 9.8% of the women being Rh-negative.

These results concur with previous studies from a different geographical region, Yola, Nigeria, where the O blood group was found in 47.7% of the people, followed by A (26.6%), B (22.6%), and AB (3.5%) blood groups^4^.

Another study from Northern Ethiopia also reported a comparable prevalence of different blood groups (O: 41.5%, A: 28%, B: 25%, and AB: 5.5%)^7^.

A similar pattern of results regarding the Rh blood groups was observed in another retrospective study conducted in Konya, Turkey, where the majority of pregnant females had Rh+ blood (90%)^5^. Also, a study reporting on the prevalence of Rh blood groups described that only 15.8% of pregnant women attending antenatal care in Libya were Rh-negative, and the negativity was higher in O and A blood groups (5.83%)^2^.

Notably the prevalence of RhD-positive group O patients was 90.1% compared to RhD negative group O patients (9.9%). This is in contrast to a prevalence of 46.6% for RhD positive type O patients reported out of Nigeria^4^; similar results were observed for other blood groups as well. However, in our study, the incidence of blood group O was 38.9%, which was lower than the prevalence of 41.1% in pregnant Ethiopian women receiving antenatal care at the Sodo Health Center^7^. Furthermore, the prevalence of Rh+ type was generally higher than in most studies conducted across different locations and countries. According to a study conducted in Northern Ethiopia, the negativity rate for Rh antigen was higher in the O blood group (3.7%)^9^, whereas another Ethiopian study revealed that O-Rh− blood group was the most predominant (42.2%)^9^. Due to the variations in ethnic and genetic characteristics among populations, these differences can be explained by how blood groups are determined.

A study conducted in Bangladesh using a smaller sample size of 430 participants reported the O blood group to be the most prevalent (35.8%). Notably, they described that the B blood group was the second most common type (29.3%), followed by the A (19.3%), which is in contrast with our results (A more common than B). Also, the prevalence of the AB blood group was higher than our results (15.6%), as was the percentage of the Rh+ category (97.9%)^10^. Likewise, another retrospective case–control study conducted on a sample of 4516 pregnant women who delivered between December 1, 2014, and March 31, 2016, at the Carmel Lady Davis Medical Center, Haifa, Israel, reported that the most prevalent blood group was the A blood group (38.6%), followed by the O blood group which had a nearly equal percentage of 35.3%. While these numbers are in stark contrast with our results, the prevalence of the B (18.3%) and AB (7.8%) blood groups was comparable to our results^11^. Our general distribution appears to be different from that in the European population, Volken et al. reported in their study of the distribution of blood groups in Switzerland, that A is the commonest blood group, followed by the O blood group (45.2% and 40.9%), while the B and AB groups are much less (9.8%, 4.1%), as for, the Rh status it also differed; their population has a higher Rh-negative percentage compared to ours (15%)^12,13^.

Although our sample size was greater than the previous studies, we had only included pregnant women from one hospital in Amman, Jordan. Also, since we did not involve non-pregnant women, men, and children in our study, the results cannot be generalized to the whole Jordanian population.

In medical science, even if something is less commonly found, it demands equal attention and caution, i.e., in the case of blood transfusions or pregnancies, it is strictly unadvisable to assume and treat the patient as if they have Rh+ blood without proper investigations. A good and competent clinician must always be cautious and order appropriate testing. In the case of a suspected non-sensitized Rh– mother, proper testing for the Rh blood group type and indirect Coombs test must be done, and caution exercised before giving anti-D immunoglobulin injections at the gestational age of 28 weeks or within 72 h of delivery of an Rh+ fetus or immediately after any procedure done during pregnancy which may involve a mixing of maternal and fetal blood, such as abortion, ectopic pregnancy, amniocentesis. Other benefits of determining blood group types include awareness and information regarding their blood group type in women of reproductive age.

## Conclusions

The majority of pregnant females undergoing childbirth at the antenatal care clinic in the Jordan University Hospital had O and Rh (D) positive blood groups. Determining the blood group type accurately helps eliminate the critical consequences of both ABO and Rh incompatibility and offers clinicians an opportunity to take timely prophylactic measures. This study reflects the population blood group distribution. We aim to be a valuable member of an international donor bank as these results will help to draw a worldwide blood donation map.

## Data Availability

The data and material for the current study are available from the corresponding author upon reasonable request.

## Abbreviations

HDFN: Hemolytic disease of the fetus and newborn
GDM: Gestational diabetes
HELLP: Hemolysis, elevated liver enzymes and low platelets
IUGR: Intrauterine growth restriction
VTE: Venous thromboembolism
EDTA: Ethylene diamine tetra acetic acid

## References

[1] Sajan R (2021). Frequency of ABO blood group in pregnant women and its correlation with pregnancy-related complications. Cureus.

[2] Azab EA, Albasha MO, Elhemady SY (2017). Haematological parameters in pregnant women attended antenatal care at Sabratha Teaching Hospital in Northwest, Libya. Am. J. Lab. Med..

[3] Hroob AMA, Saghir SAM, Almaiman AA (2020). Prevalence and association of transfusion transmitted infections with ABO and Rh blood groups among blood donors at the National Blood Bank, Amman, Jordan. Medicina.

[4] Ajayi DO, Omon EA, Orekoya A, Oludare O (2022). Haemoglobin genotype, ABO and rhesus blood group pattern among students of Bamidele Olumilua University of Education, Science and Technology Ikere, Ekitis state, Nigeria. Int. J. Res. Med. Sci..

[5] Gündem NS, Ataş E (2019). Distribution of ABO and Rh Blood Groups among patients admitted to a Gynaecology, Obstetrics and Children Hospital in Konya, Turkey. J. Clin. Diagn. Res..

[6] Delaney M, Matthews DC (2015). Hemolytic disease of the fetus and newborn: managing the mother, fetus, and newborn. Hematology.

[7] Chanko KP (2020). Frequency of ABO blood group and Rh (D) negative mothers among pregnant women attending at Antenatal Care Clinic of Sodo Health Center, SNNPR, Ethiopia. Am. J. Clin. Exp. Med..

[8] Saqlain N, Ahmed A, Fateen T, Ahmed N (2016). D antigen: Chances of finding weak D antigen and re-evaluation of its clinical significance as a routine blood bank procedure. Prof Med J..

[9] Alemu M, Abrha G, Bugssa G, Tedla K (2014). Frequency of ABO and Rh (D) blood groups and hemoglobin threshold among pregnant women in family guidance association, Mekelle Model Clinic, North Ethiopia. Int. J. Pharm. Sci. Res..

[10] Akter K (2020). Distribution of blood group among pregnant women in a rural area of Bangladesh. J. Xiangya. Med..

[11] Ali-Saleh M, Lavie O, Abramov Y (2019). Evaluation of blood type as a potential risk factor for early postpartum hemorrhage. PLoS ONE.

[12] Dean, L. *Blood Groups and Red Cell Antigens* (National Center for Biotechnology Information (US), 2005). https://www.ncbi.nlm.nih.gov/books/NBK2261/.

[13] Volken T (2017). Blood group distribution in Switzerland: A historical comparison. Transfus. Med. Hemother..

